# Association of preoperative sarcopenia with the long-term prognosis of patients with bladder cancer undergoing radical cystectomy

**DOI:** 10.1007/s00432-024-05705-6

**Published:** 2024-04-03

**Authors:** Sangmin Lee, Youngjoon Yoon, Jungyo Suh, Dalsan You, Bumsik Hong, Jun Hyuk Hong, Hanjong Ahn, In Gab Jeong, Bumjin Lim

**Affiliations:** 1 Department of Urology, Asan Medical Center, University of Ulsan College of Medicine, 88 Olympic-ro 43-gil, Songpa-gu, Seoul, 05505 Korea; 2 Department of Radiation Oncology, Asan Medical Center, University of Ulsan College of Medicine, 88 Olympic-ro 43-gil, Songpa-gu, Seoul, 05505 Korea

**Keywords:** Urinary bladder neoplasms, Cystectomy, Sarcopenia, Prognosis, Survival analysis

## Abstract

**Purpose:**

This retrospective study aimed to assess the correlation between preoperative sarcopenia and long-term oncologic outcomes in patients undergoing radical cystectomy for bladder cancer.

**Methods:**

We included 528 patients who underwent radical cystectomy for bladder cancer between 2000 and 2010 at Asan Medical Center, Seoul, Korea. Preoperative skeletal muscle mass was quantified by analyzing computed tomography images at the third lumbar vertebra. Sarcopenia was defined based on the skeletal muscle index. We evaluated various clinical and pathological factors to analyze the association between sarcopenia and long-term oncologic outcomes.

**Results:**

The median follow-up time was 104 months. Sarcopenia was identified in 37.9% of the patients. Although no significant differences were observed in traditional pathological factors between the sarcopenic and non-sarcopenic groups, sarcopenia was significantly associated with worse oncologic outcomes. Compared to the non-sarcopenic groups, the sarcopenic group had lower overall survival rates (52.0% vs. 67.1% at 5 years, 35.5% vs. 52.7% at 10 years) and higher cancer-specific mortality (63.3% vs. 74.3% at 5 years, 50.7% vs. 67.4% at 10 years). Multivariable Cox regression analysis demonstrated that sarcopenia was an independent predictor of cancer-specific survival (hazard ratio: 1.49, 95% confidence interval: 1.11–2.01, p = 0.008), alongside body mass index, tumor stage, lymph node metastasis, and lymphovascular invasion.

**Conclusion:**

Sarcopenia was significantly associated with poor cancer-specific survival in patients undergoing radical cystectomy for bladder cancer. Detecting sarcopenia may assist in preoperative risk stratification and long-term management after radical cystectomy.

## Introduction

Bladder cancer (BCa) is the 10th most diagnosed cancer globally, with around 573,000 new cases and 213,000 deaths reported in 2020. Moreover, BCa is more prevalent among men than women, ranking as the 6th most common cancer and the 9th leading cause of cancer-related deaths in men (Sung et al. 2021). Approximately 25% of BCa cases are identified as muscle-invasive bladder cancer (MIBC) at the time of diagnosis (Smith et al. 2014). The gold standard treatment for patients with MIBC is radical cystectomy (RC) with pelvic lymph node dissection, combined with neoadjuvant chemotherapy (NAC) (Chang et al. 2017). However, a significant number of patients experience disease recurrences and progressions, resulting in approximately 65% 5-year cancer-specific survival (CSS) rate after RC (Nuhn et al. 2012). Despite advancements in understanding BCa’s pathophysiology and the introduction of new adjuvant therapies, BCa remains a challenge due to high mortality rates. In addition to traditional tumor, node, metastasis (TNM) staging, several pathologic factors, including tumor grade, lymphovascular invasion, and positive surgical margins are acknowledged as predictors of poor prognosis in BCa after RC (Hong et al. 2017; Kim et al. 2014; Zhang et al. 2019). There is a study suggesting that advanced age is a significant predictor for CSS in patients with BCa (Zhang et al. 2019). Still, a lack of studies exist on clinical factors that significantly influence the prognosis of patients with BCa after RC. Hence, the identification of clinical factors capable of predicting prognosis in patients with BCa could greatly assist in determining individualized treatment options.

Sarcopenia is a progressive and systemic skeletal muscle disorder characterized by severe wasting of skeletal muscle and strength/function (Cruz-Jentoft et al. 2019). Although the exact pathophysiological relationship between cancer and sarcopenia remains unclear, it is known that cancer triggers sarcopenia through various pathways. Cancer-related symptoms or neuroendocrine changes often exacerbate anorexia (Fearon et al. 2012), while proinflammatory cytokines (tumor necrosis factor-alpha, interleukin (IL)-6, and IL-1, etc.) contribute to muscle wasting (Coletti et al. 2005; Petruzzelli et al. 2014). Sarcopenia can serve as a clinical factor, indicating the systemic impact of cancer and potentially playing a role as a prognostic factor.

Many studies have demonstrated that oncologic outcomes may depend on sarcopenia as well as conventional tumor-related factors in various cancers (Caan et al. 2018; Go et al. 2016; Martin et al. 2013). Over the past decade, several studies demonstrated that sarcopenia was identified as a potential predictor of oncological outcomes in BCa in addition to postoperative complications (Engelmann et al. 2023; Mayr et al. 2018a; Mayr et al. 2018b; Psutka et al. 2014). However, previous studies were limited by the small sample size or relatively short-term follow-up period, which limits the wide use of sarcopenia as a factor in predicting oncological outcomes in patients undergoing RC for BCa. Therefore, we investigated the association of sarcopenia and skeletal muscle index (SMI) with the long-term oncologic outcomes of patients with BCa after RC using a large retrospective cohort from a tertiary referral center.

## Materials and methods

### Patient population

The present study was conducted with the approval and oversight of the institutional review board of Asan Medical Center, Seoul, Korea (IRB approval number 2024-0083), and the need to obtain informed patient consent was waived. We retrospectively identified 584 consecutive patients who underwent RC for BCa at Asan Medical Center, Seoul, Korea, between 2000 and 2010. Of the initial 584 patients, 45 were excluded due to unavailable digital files, six had computed tomography (CT) scans that did not cover the third lumbar vertebra (L3) level, two had metal instruments at the spinal level, two had undergone prior radiotherapy, and one had a history of polio. After these 56 cases were excluded, a total of 528 patients were included in the study.

All patients underwent preoperative examination including chest radiography, CT of the abdomen and pelvis, and bone scans for disease staging. Patient demographics and clinical characteristics were evaluated. Genitourinary pathologists determined tumor grades based on the 2004 World Health Organization grading system, and pathological staging was performed according to the Seventh Edition of the American Joint Committee on Cancer/Union Internationale Contre le Cancer TNM classification (Montironi and Lopez-Beltran 2005).

In the absence of recurrence or metastasis, patients underwent routine testing every 3 months during the first year, every 6 months for the next 5 years, and annually after that. Routine tests comprised blood laboratory tests, urine analysis, culture, cytology, and imaging studies, including chest radiography, CT of the abdomen and pelvis, and bone scans.

### Preoperative measurement of skeletal muscle mass and definition of sarcopenia

Skeletal muscle mass was evaluated through the measurement of the cross-sectional areas of skeletal muscles (SMA, cm^2^) at the L3 level, where both vertebral spines were observable. Measurements were conducted using AsanJ-Morphometry^TM^, specialized software based on ImageJ (NIH, Bethesda, MD, USA), designed for quantifying tissue measurements (available at http://datasharing.aim-aicro.com/morphometry). The CT Hounsfield unit (HU) threshold for identifying skeletal muscle was set at −30 to 150 HU. The assessed muscles included the psoas, paraspinal, transversus abdominis, rectus abdominis, quadratus lumborum, and internal and external obliques in the specified axial images. Additionally, SMA was normalized for height (m^2^) and reported as the SMI at L3 (cm^2^/m^2^). Sarcopenia was defined as an SMI < 43 cm^2^/m^2^ for men with a body mass index (BMI) < 25 kg/m^2^, an SMI < 53 cm^2^/m^2^ for men with a BMI ≥ 25 kg/m^2^, and an SMI < 41 cm^2^/m^2^ for women, according to the criteria by Martin et al (Martin et al. 2013).

### Statistical analyses

Patients were categorized into sarcopenic and non-sarcopenic groups. The Wilcoxon–Mann–Whitney U test and Pearson’s chi-square test were used to compare continuous and categorical variables respectively. To investigate the association between SMI and age/BMI, linear regression analyses were conducted within the entire cohort and stratified by gender. Overall survival (OS) and CSS were analyzed and compared between groups using the Kaplan–Meier method and the log-rank test. Associations of individual clinical and pathologic factors with OS and CSS were assessed using the Cox proportional hazards regression model. All statistical analyses were performed using R Statistical Software (version 4.3.1, R Core Team, Vienna, Austria), and p-values < 0.05 were considered significant.

## Results

Of the 528 patients, 200 (37.9%) were classified as sarcopenic. The baseline clinical and pathological characteristics are presented in Table 1. The sarcopenic group was older on average (68 vs. 63 years, p < 0.001) and had a higher proportion of women (20.0% vs. 7.3%, p < 0.001) compared to the non-sarcopenic group. The prevalence of hypertension was high in patients with sarcopenia (36.5% vs. 26.2%, p = 0.012), and a significantly large proportion had a poor Eastern Cooperative Oncology Group (ECOG) (≥2) performance status (6.0% vs. 2.1%, p = 0.021). Additionally, a high number of patients in the sarcopenic group underwent urinary diversion with an ileal conduit (36% vs. 26%, p = 0.024). However, no significant differences were observed between the groups in terms of other clinical characteristics and all pathological variables, including TNM stage, lymphovascular invasion, presence of carcinoma in situ, and surgical margins (p > 0.05 for all).

**Table 1:** Patient characteristics of non-sarcopenic and sarcopenic patients

Characteristic	All	Non-sarcopenic	Sarcopenic	p-value^*a*^
N (%)	528(100)	328 (62.1)	200 (37.9)	
Age (years, IQR)	65 (58, 71)	63 (55, 69)	68 (62, 72)	<0.001
Male gender (%)	464 (87.9%)	304 (92.7%)	160 (80.0%)	<0.001
Height (cm, IQR)	165.5 (160.0, 170.0)	165.5 (160.2, 169.5)	165.4 (159.7, 170.6)	0.996
Weight (kg, IQR)	64.3 (58.0, 71.5)	64.6 (59.0, 70.9)	63.0 (56.6, 73.0)	0.437
BMI (kg/m^2^, IQR)	23.8 (21.5, 25.9)	23.8 (21.9, 25.8)	24.0 (21.2, 26.1)	0.748
BMI category (%)				0.072
Underweight	21 (4.0%)	9 (2.7%)	12 (6.0%)	
Normal	187 (35.4%)	109 (33.2%)	78 (39.0%)	
Overweight	262 (49.6%)	175 (53.4%)	87 (43.5%)	
Obesity	58 (11.0%)	35 (10.7%)	23 (11.5%)	
Hypertension (%)	159 (30.1%)	86 (26.2%)	73 (36.5%)	0.012
Diabetes mellitus (%)	77 (14.6%)	46 (14.0%)	31 (15.5%)	0.641
ECOG PS ≥2 (%)	19 (3.6%)	7 (2.1%)	12 (6.0%)	0.021
Ileal conduit diversion (%)	157 (29.7%)	86 (26.2%)	71 (35.5%)	0.024
Pathologic T stage (%)				0.200
Less than 1	188 (35.6%)	126 (38.4%)	62 (31.0%)	
2	118 (22.3%)	75 (22.9%)	43 (21.5%)	
3	152 (28.8%)	85 (25.9%)	67 (33.5%)	
4	70 (13.3%)	42 (12.8%)	28 (14.0%)	
Lymph node metastasis (%)	134 (25.4%)	82 (25.0%)	52 (26.0%)	0.798
Presence of CIS (%)	135 (25.6%)	80 (24.4%)	55 (27.5%)	0.427
Presence of lymphovascular invasion (%)	216 (40.9%)	130 (39.6%)	86 (43.0%)	0.445
Surgical margin positive (%)	30 (5.7%)	19 (5.8%)	11 (5.5%)	0.888
Adjuvant chemotherapy (%)	135 (25.6%)	85 (25.9%)	50 (25.0%)	0.815
SMA at L3 (cm^2^, IQR)	132.7 (113.8, 148.2)	140.5 (127.8, 156.4)	114.0 (97.5, 131.1)	<0.001
L3 SMI (cm^2^/m^2^, IQR)	48.0 (42.7, 53.7)	51.8 (46.5, 57.0)	40.9 (37.6, 47.3)	<0.001

*IQR* interquartile range, *BMI* Body mass index, *ECOG* Eastern Cooperative Oncology Group, *PS* Performance status, *CIS* Carcinoma in situ, *SMA* Skeletal muscle area, *SMI* Skeletal muscle index

^a^Wilcoxon rank sum test; Pearson’s Chi-squared test

**Fig. 1:**
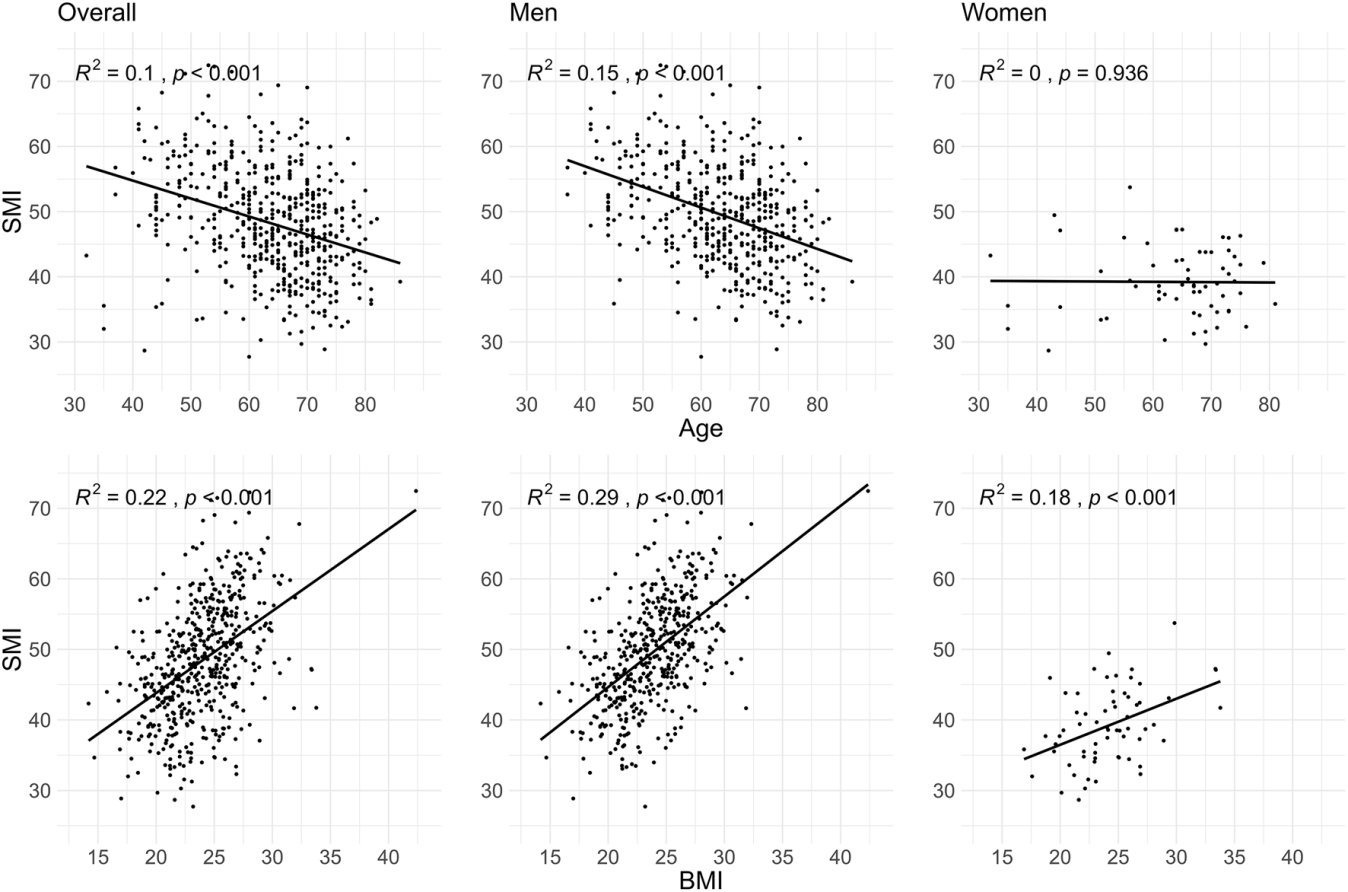
Linear regression analysis of skeletal muscle index with age and body mass index: overall and by gender

Among the 528 patients, the median SMI was 48.0 cm^2^/m^2^. The median SMI among men in the study cohort was 49.1 cm^2^/m^2^ (interquartile range (IQR), 44.5–54.8 cm^2^/m^2^), whereas it was 38.6 cm^2^/m^2^ (IQR, 35.2–43.1 cm^2^/m^2^) for women. Linear regression analysis displayed a significant correlation between SMI and age (R^2^ = 0.102, p < 0.001) (Figure 1). However, this correlation was almost negligible among women, indicating a minimal relationship between SMI and age in women (p > 0.05). In the linear regression analysis between BMI and SMI a significant positive correlation was observed regardless of gender (R^2^ = 0.22, p < 0.001).

**Fig. 2:**
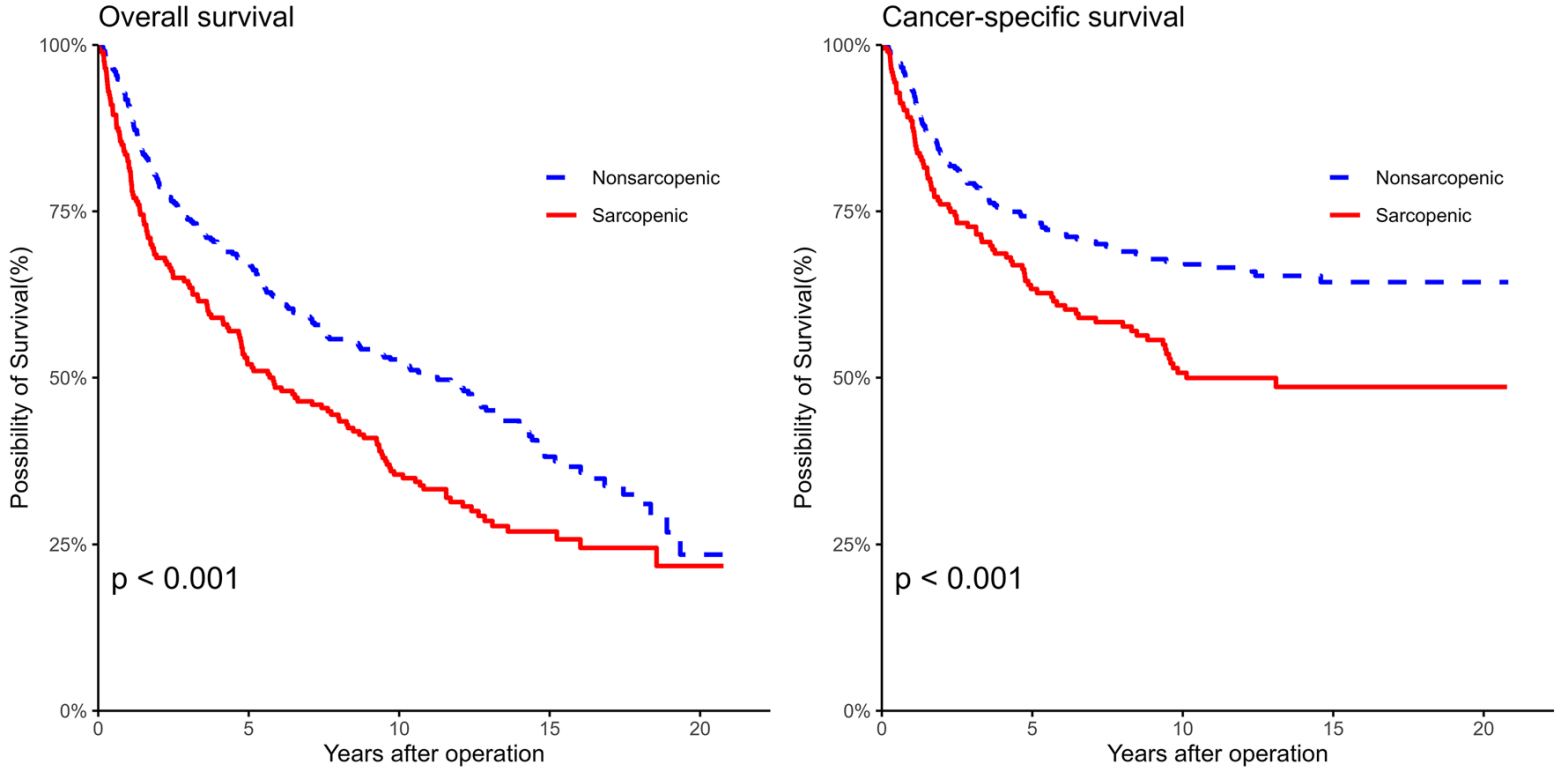
Kaplan–Meier curve of overall and cancer-specific survival in sarcopenic and non-sarcopenic patients

The median overall follow-up of the patients was 104 months (IQR, 24–154 months). During the median follow-up period, 264 (50.0%) patients died, with 176 (33.3%) of these deaths attributed to BCa. The 5-year and 10-year OS rates for the sarcopenic group were 52.0% and 35.5%, respectively, significantly lower than those of the non-sarcopenic group (67.1% and 52.7%, p < 0.00043) (Figure 2). Similarly, the 5-year and 10-year CSS rates were significantly lower in the sarcopenic group (63.3% vs. 74.3% at 5 years, 50.7% vs. 67.4% at 10 years; p = 0.00086) than in the non-sarcopenic group.

In the Cox regression analysis for CSS (Table 2), sarcopenia emerged as a significant prognostic factor in both univariable (hazard ratio (HR): 1.61, 95% confidence interval (CI): 1.21–2.14, p < 0.001) and multivariable analyses (HR: 1.49, 95% CI: 1.11–2.01, p = 0.008). Additional significant factors associated with CSS included BMI, pathologic T stage (≥3), lymph node metastasis, and lymphovascular invasion. However, in the overall survival analysis (Table 3), while sarcopenia was significant in the univariable analysis (HR: 1.47, 95% CI: 1.18–1.82, p < 0.001), it did not remain significant in the multivariable analysis (HR: 1.23, 95% CI: 0.98–1.54, p = 0.071). For OS, old age, low BMI, diabetes mellitus, poor ECOG performance status (≥2), high pathologic T stage (≥3), and lymph node metastasis were significant factors predicting increased all-cause mortality.

**Table 2:** Univariable and multivariable Cox regression analysis of factors associated with cancer-specific survival in patients with bladder cancer undergoing radical cystectomy

	Univariable analysis			Multivariable analysis		
	HR^*a*^	95% CI^*a*^	p-value	HR^*a*^	95% CI^*a*^	p-value
Age (year)	1.02	1.01, 1.04	0.003	1.01	1.00, 1.03	0.086
Gender (Male versus female)	1.25	0.83, 1.88	0.281	–	–	
BMI (kg/m^2^)	0.92	0.87, 0.96	<0.001	0.94	0.90, 0.99	0.011
Hypertension	1.04	0.77, 1.42	0.779	–	–	
Diabetes mellitus	1.21	0.83, 1.78	0.326	–	–	
ECOG PS (≥2 versus <2)	1.18	0.52, 2.66	0.691	–	–	
Diversion type (Neobladder versus Ileal conduit)	2.15	1.61, 2.88	<0.001	1.13	0.82, 1.57	0.458
Pathologic T stage				–	–	
Less than 1	–	–		–	–	
2	1.68	1.04, 2.71	0.034	1.20	0.73, 1.99	0.473
3	4.35	2.91, 6.52	<0.001	2.29	1.44, 3.62	<0.001
4	6.77	4.34, 10.5	<0.001	3.23	1.90, 5.51	<0.001
Lymph node metastasis	4.24	3.18, 5.66	<0.001	2.74	1.86, 4.04	<0.001
Presence of CIS	1.02	0.74, 1.41	0.908	–	–	
Lymphovascular invasion	3.48	2.60, 4.67	<0.001	1.57	1.11, 2.24	0.011
Surgical margin positive	1.52	0.90, 2.58	0.117	–	–	
Adjuvant chemotherapy	2.65	1.98, 3.53	<0.001	0.89	0.60, 1.31	0.560
L3 SMI (cm^2^/m^2^)	0.96	0.95, 0.98	<0.001	–	–	
Sarcopenia	1.61	1.21, 2.14	<0.001	1.49	1.11, 2.01	0.008

^*a*^*HR* Hazard Ratio, *CI* Confidence Interval

*BMI* Body mass index, *ECOG* Eastern Cooperative Oncology Group, *PS* Performance status, *CIS* Carcinoma in situ, *SMI* Skeletal muscle index

**Table 3:** Univariable and multivariable Cox regression analysis of factors associated with overall survival in patients with bladder cancer undergoing radical cystectomy.

	Univariable analysis			Multivariable analysis		
	HR^*a*^	95% CI^*a*^	p-value	HR^*a*^	95% CI^*a*^	p-value
Age (year)	1.05	1.04, 1.06	<0.001	1.04	1.03, 1.06	<0.001
Gender (Male versus female)	1.05	0.76, 1.47	0.757	–	–	–
BMI (kg/m^2^)	0.92	0.89, 0.95	<0.001	0.94	0.90, 0.97	<0.001
Hypertension	1.09	0.87, 1.38	0.447	–	–	–
Diabetes mellitus	1.43	1.09, 1.90	0.011	1.67	1.25, 2.24	<0.001
ECOG PS (≥2 versus <2)	2.24	1.37, 3.66	0.001	1.75	1.06, 2.91	0.030
Diversion type (Neobladder versus Ileal conduit)	2.09	1.67, 2.60	<0.001	1.05	0.81, 1.35	0.722
Pathologic T stage						
Less than 1	–	–	–	–	–	–
2	1.46	1.07, 1.99	0.016	1.14	0.82, 1.58	0.434
3	2.80	2.12, 3.69	<0.001	1.92	1.40, 2.63	<0.001
4	3.56	2.56, 4.96	<0.001	2.20	1.49, 3.27	<0.001
Lymph node metastasis	3.04	2.42, 3.83	<0.001	2.77	2.02, 3.79	<0.001
Presence of CIS	0.93	0.72, 1.19	0.565	–	–	–
Lymphovascular invasion	2.25	1.82, 2.80	<0.001	1.27	0.98, 1.66	0.074
Surgical margin positive	1.06	0.67, 1.68	0.805	–	–	–
Adjuvant chemotherapy	1.65	1.31, 2.08	<0.001	0.73	0.53, 1.01	0.057
L3 SMI	0.97	0.96, 0.98	<0.001	–	–	–
Sarcopenia	1.47	1.18, 1.82	<0.001	1.23	0.98, 1.54	0.071

^*a*^*HR* Hazard Ratio, *CI* Confidence Interval

*BMI* Body mass index, *ECOG* Eastern Cooperative Oncology Group, *PS* Performance status, *CIS* Carcinoma in situ, *SMI* Skeletal muscle index

## Discussion

This study evaluates the relationship between preoperative sarcopenia and long-term oncologic outcomes in patients undergoing RC for BCa. Despite no significant differences in traditional pathologic factors between sarcopenic and non-sarcopenic groups, we observed significant associations between sarcopenia and poor oncologic outcomes. Specifically, low OS rates and increased cancer-specific mortality were observed in the sarcopenic group, emphasizing the potential impact of sarcopenia on long-term survival in patients with BCa post-RC. Sarcopenia was a significant independent predictor of poor CSS in patients who underwent RC for BCa. However, in our multivariable analyses, while sarcopenia demonstrated a notable HR for OS, it did not reach statistical significance. This suggests that while sarcopenia may play a role, the influence of the condition on long-term OS might be intertwined with other clinical factors such as age, BMI, performance status, diabetes, and traditional pathologic stage.

Sarcopenia is defined as a muscle disease characterized by a qualitative or quantitative decline in muscles and diminishing muscle strength (Cruz-Jentoft et al. 2019). Not only attributed to aging, but sarcopenia can also be caused or exacerbated by inflammatory conditions, malnutrition, inactivity, etc (Jimenez-Gutierrez et al. 2022; Steffl et al. 2017). Many studies have investigated the association between sarcopenia and cancer prognosis, yet the exact mechanisms remain unclear. In most of these studies, sarcopenia is primarily defined by the low quantity of muscle mass; however, achieving uniformity in the methods used to measure muscle quantity and establish optimal cutoff values remains a challenge. The measurement of muscle area using CT is advantageous as the method applies to most patients, given that nearly all undergo abdominal CT scans at the time of diagnosis. Additionally, CT images focusing on a particular lumbar vertebral reference point (L3) demonstrated a significant correlation with overall body muscle mass (Mourtzakis et al. 2008).

In this study, we defined sarcopenia using the Martin criteria (Martin et al. 2013). These criteria were developed from a cohort of 1473 patients with cancer using optimal stratification of low survival, establishing them as the most accurate CT-based classification for sarcopenia. The same criteria have been employed in recent studies to investigate the association between oncologic outcomes and sarcopenia in patients with BCa who underwent RC. In a large multicenter study with patients undergoing RC for BCa, sarcopenia was demonstrated to be an independent predictor for OS and CSS (HR: 1.42, 95% CI: 1.00–2.02, p = 0.048) (Mayr et al. 2018b). However, this study was limited by the relatively short median follow-up of patients (35 months; IQR, 20–58 months), possibly insufficient for assessing long-term survival. In studies conducted in Japan, the presence of sarcopenia displayed a significant association with poor CSS (Hirasawa et al. 2016) (HR: 2.3, 95% CI: 1.2–4.4, p = 0.015) and OS (Miyake et al. 2017) (HR: 2.2, 95% CI: 1.1–4.6, p = 0.03). Both these Japanese studies demonstrated rates of sarcopenia (48% and 25%) similar to our study (37.9%). However, they were constrained by small sample sizes and short follow-up periods.

Engelmann et al. conducted a retrospective single-center study with 657 patients to assess the impact of various body composition parameters on mortality in BC patients post-RC (Engelmann et al. 2023). This study confirmed the impact of sarcopenia, as defined by the Martin criteria, and broadened its scope to explore the effects of sarcopenia identified using various criteria, low psoas muscle index, and myosteatosis, along with their correlations with OS and CSS. The significance of this study is underscored by its large sample size and a substantial median follow-up period (40 months; IQR 15–76 months), making it a significant contribution to the field. Similarly, our research enrolled a considerable cohort aligns with the findings of Engelmann et al. Beyond these similarities, our study distinguishes itself by exploring the implications of sarcopenia over an even longer follow-up period, thereby enriching our understanding of its prognostic significance in patients with BCa after RC.Our findings align with prior studies demonstrating a high prevalence of sarcopenia in older individuals, as evidenced by the advanced age observed in the sarcopenic group. Interestingly, this study revealed a statistically significant negative correlation between age and skeletal muscle mass index (SMI) in men. In contrast, such a correlation was not observed in women, which may be attributed to the smaller sample size of female patients potentially limiting the detection of an association. A study focusing on healthy adults has shown a linear correlation between SMI and age in women, suggesting that our findings may differ due to the specific characteristics of our study population (Kong et al. 2022). The sarcopenic group displayed differences compared to the non-sarcopenic group in the prevalence of hypertension, poor ECOG performance status, and specific surgical interventions like ileal conduit diversion. The differences in characteristics between these two groups appear to be the result of the systemic impact of sarcopenia. However, except for poor ECOG performance status being a significant predictor in the multivariable analysis for OS, these characteristics did not significantly impact CSS or OS.

Despite the valuable insights gained from this study, several limitations should be acknowledged. First, the retrospective nature of this study entails inherent limitations. Although attempting to assess a considerable number of patients, retrospective analyses can be affected by selection bias and missing data, potentially influencing the outcomes. Second, as this study was conducted in a single center, the generalizability of the findings to broad populations or healthcare settings might be limited, potentially impacting the external validity of the results. To overcome these limitations, a large multicenter prospective study should be conducted. Third, the study duration, covering the years 2000–2010, could introduce biases due to alterations in diagnostic techniques and treatment modalities. The standard treatment involving NAC before RC for patients with MIBC became established after the 2010s. As a result, only 17 individuals (3.2%) in our study cohort underwent NAC. Further investigations are necessary to explore the influence of sarcopenia in patients undergoing NAC. Finally, while the Martin criteria were employed to define sarcopenia, variations in cutoff values and methods used across different studies defining sarcopenia might introduce inconsistencies in the comparison and interpretation of findings.

Notwithstanding these limitations, our study is, to the best of our knowledge, the most extensive in duration, providing valuable insights into the role of sarcopenia as a prognostic factor for CSS in patients with BCa post-RC. Future prospective studies could enhance our understanding of the complex interrelations between sarcopenia and oncologic outcomes, leading to tailored treatment strategies for patients with BCa.

## Conclusions

This study investigated the relationship between preoperative sarcopenia and long-term oncologic outcomes in patients undergoing RC for BCa. Sarcopenia was an independent risk factor for CSS in patients undergoing RC for BCa. Identification of sarcopenia may assist in preoperative risk stratification and long-term management after RC.

## Data Availability

The data sets generated during and/or analyzed during the current study are available from the corresponding author on reasonable request.
